# Ultrafast plasmonic photoemission in the single-cycle and few-cycle regimes

**DOI:** 10.1038/s41598-022-07259-4

**Published:** 2022-03-10

**Authors:** G. Zs Kiss, P. Földi, P. Dombi

**Affiliations:** 1 Wigner Research Centre for Physics, Konkoly-Thege M. u. 29-33, Budapest, 1121 Hungary; 2 Department of Theoretical Physics, University of Szeged, Szeged, 6720 Hungary; 3 ELI-ALPS Research Institute, ELI-HU Non-Profit Ltd., Wolfgang Sandner u. 3, Szeged, 6728 Hungary

**Keywords:** Nanophotonics and plasmonics, Ultrafast photonics

## Abstract

Due to the highly increased interest in the development of state-of-the-art applications of photoemission in ultrafast electron microscopy, development of photocathodes and many more applications, a correct theoretical understanding of the underlying phenomena is needed. Within the framework of the single active electron approximation the most accurate results can be obtained by the direct solution of the time-dependent Schrödinger equation (TDSE). In this work, after a brief presentation of a numerically improved version of a mixed 1D-TDSE method, we investigated the characteristics of electron spectra obtained from the surface of metal nanoparticles irradiated with ultrashort laser pulses. During our investigation different decay lengths of the plasmonic-enhanced incident field in the vicinity of the metal were considered. Using the simulated spectra we managed to identify the behavior of the cutoff energy as a function of decay length in the strong-field, multiphoton and transition regimes.

## Introduction

Developments in laser physics and nanotechnology opened new horizons in the last few decades in understanding the nature of light–matter interaction processes at ultrafast time scales on the nanoscale^1^. It was shown recently that by measuring the spectra of laser induced photoelectrons from nanotips, and nanostructured surfaces, the development of electron sources for ultrafast electron microscopy^1–3^ and high-brightness photocathodes^4–6^ can be performed. In addition, by analyzing the characteristic shapes of the acquired electron spectra one can differentiate between various ultrafast light–metal interaction mechanisms, which can be later deployed to achieve different applications.

By measuring the photoemission spectra acquired from the surface of the laser-irradiated nanoparticles one can access information about the local electric fields on the nanoscale^7,8^, that is a major interest, since sthe incident fields in certain circumstances can be enhanced by order of magnitudes by the local plasmonic oscillations^9–12^.

For the deeper understanding of the underlying phenomena^13–19^, accurate theoretical methods that are accessible with the current computational resources are needed. The most accurate results are based on the direct numerical solution of the time-dependent Schrödinger equation (TDSE), however, typically these type of calculations are largely time-consuming, and practically unfeasible for many-electron systems of metal targets. Considering this, the many electron-system investigations are usually the subject of time-dependent density functional (TD-DFT) theory calculations. However, if one wants to decipher the single-electron response to the applied electric field (e.g., the field-induced electron dynamics), for certain circumstances, such as when the incident laser field is linearly polarized along a symmetry axis of an investigated system (e.g. along the direction of a nanotip, or of the edge of a nanotriangle) the solution of the 1D-TDSE can also deliver reliable and good results within an accessible CPU time.

Here, we analyze photoelectron emission induced by femtosecond laser pulses with 1D-TDSE in nanolocalized electromagnetic fields. We show characteristic features of the spectra with increasing pulse length and laser intensity. We highlight the correspondence of the electron spectra and the wave-function evolution, as well.

The manuscript is constructed as follows. In “The theoretical method and numerics” section, a brief general description of a mixed, Split-Operator and Crank–Nicolson TDSE approach together with some important numerical aspects are presented. In “Photoelectron spectra and wavefunctions” section, we present and discuss the shape of the final photoelectron spectra and electronic wavefunctions obtained for different pulse lengths and values of the Keldysh-paramater. The final section is dedicated to the investigation of the field-decay effect on the spectra in the vicinity of the nanotargets.

Throughout this paper atomic units (at.u.) are used ($\hbar =m_{e}=e=1$), and for the incident laser fields we used Gaussian pulses having the same central wavelength $\lambda$ = 800 nm (IR pulse) but different peak intensities and pulse lengths.

## Theoretical method and numerics

It was previously shown in preceding works^20^ that photoelectrons induced by laser pulses from different plasmonic nanoparticles have the larger kinetic energies the closer to edges of the nanotargets they escaped from. This observation can be explained by the fact that the edges of a nanorods/nanotriangles, or the tips of a nanotip are acting like nanoantennas for the incident EM field, i.e., under certain conditions (due to plasmonic effects induced by the field itself in the metal) the incoming *E*(*t*) electric field in the close vicinity of the target can be enhanced. The resulting locally enhanced field can be given as $E_{\mathrm {loc}}(z;t)=Q(z) E(t)$ with $Q(z) > 1$ being the enhancement factor, which decays exponentially as a function of the distance from the surface.

The dynamics of these high energy electrons that probe the enhanced electric fields in the vicinity of the nanotargets’ sharp edges are modeled by assuming a simple 1D scheme. Within this model the motion of the photoelectron points in the 0*z* direction that is perpendicular to the local surface and it is induced by the electric component of the laser field parallel to 0*z* axis. Here, 0*z* represents the symmetry axis of the laser-nanotip/edge system that is assumed obeying cylindrical symmetry around the normal of the local surface.

### The time-dependent Schrödinger equation (TDSE)

By taking into account the aforementioned considerations and in order to study more appropriately the dynamics of the laser emitted electron from the investigated nanorod (or nanotriangle) we employed the one-dimensional time-dependent Schrödinger equation (1D-TDSE) for an active electron located on the surface of the metal:

1 $$\begin{aligned} i \partial _t \Psi (z;t)= & {} {\hat{H}}(t) \Psi (z;t) \nonumber \\= & {} \left[ {\hat{T}} + {\hat{V}}(z) + {\hat{V}}_{\mathrm {le}}(z;t) \right] \Psi (z;t), \end{aligned}$$

where ${\hat{T}}=-\,\partial _z^2/2$ is the kinetic energy operator of the electron represented by the $\Psi (z;t)$ wavefunction ($\partial _{z,t}=\partial /\partial {z,t}$).

**Figure 1:**
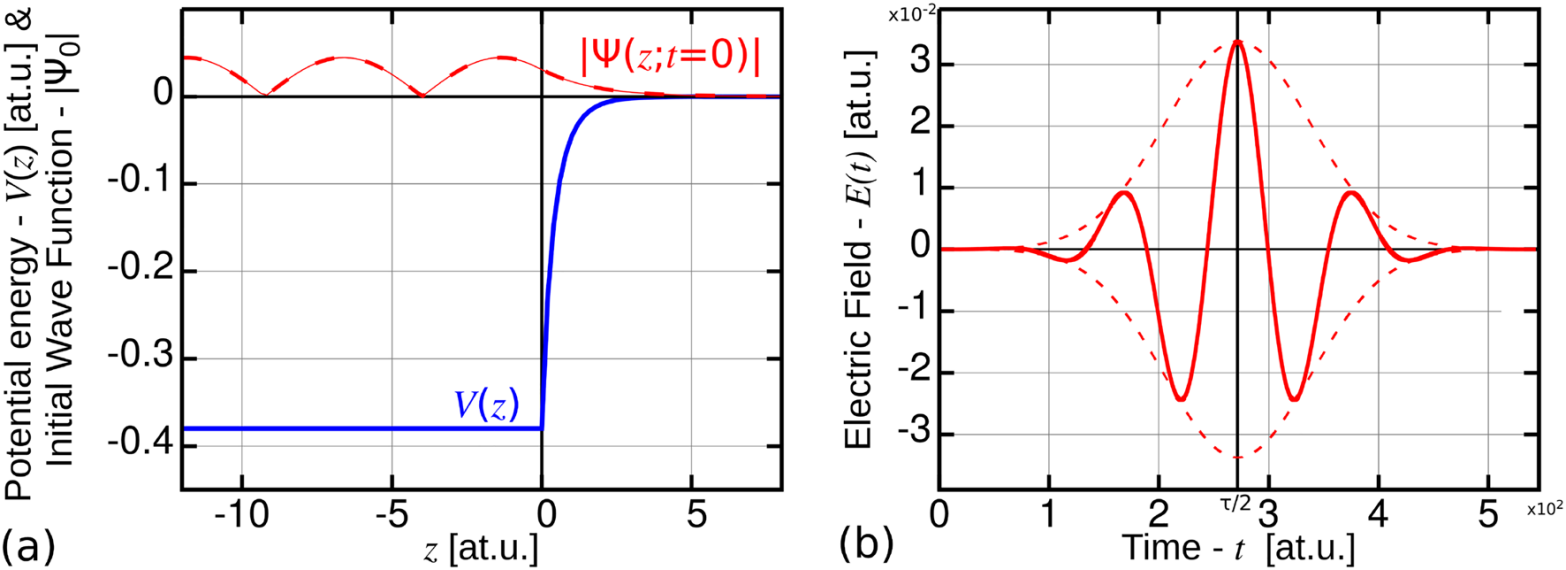
(**a**) The potential energy of the electron inside the electron bulk at the Fermi-level (lower blue curve) and its wavefunction’s absolute value (upper red curve). (**b**) The electric component of a $\lambda =800$ nm laser pulse having $n_{\mathrm {OC}}=2$ optical cycles in the FWHM of peak intensity ($\tau = 5.5$ fs = 543 at.u.; note that the time and electric field axis have units of $10^2$ and $10^{-2}$, respectively).

We consider a gas of quasi free electrons, for which the boundary of the metal means a confining potential. Using single active electron approximation, the electron experiences the following potential (Fig. 1a):

2 $$\begin{aligned} V(z)=-\frac{ \exp \big (-\beta (z+|z|)\big )}{2(z+|z|)+1/(E_\mathrm {F}+W)}, \end{aligned}$$

where $E_\mathrm {F}$ is the Fermi energy, *W* is the work function of the metal ($W_{\mathrm {Au}}\simeq 5.1$ eV in our case for gold), while the parameter $\beta$ represents the screening constant of the image potential, whose value was set to $\beta =0.6$ as showed to reproduce numerical results in good agreement with the experimental data^21^. Considering a Gaussian incident laser pulse with the electric field component (Fig. 1b):

3 $$\begin{aligned} E(t)=E_0\exp \left[ -2 \ln 2 (t/\tau _{\mathrm {p}})^2 \right] \cos (\omega _0 t + \varphi _{\mathrm {CEP}}), \end{aligned}$$

the laser–electron interaction potential was given within the dipole approximation and using its length gauge form: ${\hat{V}}_{\mathrm {le}}(z;t)$ $={\vec {{r}}} {\vec {{E}}}(t)\equiv zE(t)$. In Eq. (3) $\tau _{\mathrm {p}}=$ $\tau _{\mathrm {FWHM}}$ was the pulse duration (in the full-width at half of the maximum intensity), $\omega _0$ the central circular frequency, and $\varphi _{\mathrm {CEP}}$ represents the carrier-envelope phase of the pulse. Furthermore, we assumed that the shielding-effect of the surrounding surface electrons onto the studied electron is sufficiently large, hence, the effect of the incident *E*(*t*) field inside the metal can be neglected: $V_{\mathrm {le}}=0$ for $z<0$. The field-enhancing plasmonic effects were considered by multiplying the incoming field with the *Q*(*z*) decaying enhancement-curve, that fixes the laser-electron interaction term to ${\hat{V}}_{\mathrm {le}}(z;t)\cong z Q(z)E(t)$ $=zE_{\mathrm {loc}}(z;t)$.

From the numerical point of view, we are aware of that a 3D-TDSE calculation would provide us a much more complete study of the investigated photoemission phenomena, for instance also including the possibility of obtaining the angular energy distribution of the ejected electrons; nonetheless, such investigations concerning laser pulses with long wavelengths ($\lambda \ge 800~$ nm) and durations ($\tau \ge 2.6~$ fs) would imply unfeasible expensive computational demands. In contrast, by using the 1D procedure, the dominant photon-induced effects, including the photoemission, could be still properly investigated with a less time-consuming procedure.

### The mixed split-operator and Crank–Nicolson (SOCN) approach

For the numerical representation of the wavefunction (WF) we employed the *finite-difference* grid method. Within this approach, the potential energy terms, *V*(*z*) and $V_{\mathrm {le}}(z;t)$, are represented by diagonal matrices, while the second order differentiation operator $\partial _z^2\Psi (z)\Big |_{z=z_i}$ $\simeq [\Psi (z_{i-1})-2\Psi (z_{i})+\Psi (z_{i+1})]/\Delta z^2$ by a tridiagonal matrix. Thus, their sum, i.e., the Hamiltonian will be a sparse matrix having nonzero elements only on the three main diagonals. In the simplest approach the ${\hat{U}}(t+\delta t, t)=$ $\exp \{-i\delta t {\hat{H}}(t)\}$ time-evolution operator is temporally discretized ($\delta t \rightarrow \Delta t$) and approximated with its Taylor-expanded form ${\hat{U}}\simeq$ $\exp \{-i\Delta t {\hat{H}}(t)\}=$ $\sum _{n=0}^{n_{\mathrm {max}}}(-i\Delta t {\hat{H}})^n/n!$. The main drawback of this approach consists in the fact that the stability of the WF’s temporal propagation can be achieved only for large values of $n_{\mathrm {max}}$ terms (a radiation field dependent parameter), which would imply a large number of matrix power calculations in each time-step, while the time propagation itself remains non-unitary by definition (i.e., the WF norm, $\Vert \Psi \Vert$, is not conserved). This non-unitarity can be avoided with the use of the Crank–Nicolson (CN) procedure^24^, according to which a forward [$\Psi (t')=$ ${\hat{U}}(t',t)\Psi (t)$] and a backward [$\Psi (t')=$ ${\hat{U}}^\dagger (t',t+\Delta t)\Psi (t+\Delta t)$] propagation step to an intermediate state at time $t'=t+\Delta t/2$ is performed, and the WF is evaluated as $\Psi (t+\Delta t)\simeq$ $[{\hat{I}}+i(\Delta t/2){\hat{H}}]^{-1} [{\hat{I}}-i(\Delta t/2){\hat{H}}]\Psi (t)$ = ${\hat{U}}^{\mathrm {CN}}\Psi (t)$. In this way the unitarity is ensured. Although the CN is an unconditionally stable and unitary approach it still necessitates a considerable amount of computation time, because for each integration timestep the calculation of a matrix inverse is needed. This shortcoming becomes even more pronounced with the increase of the wavelength and/or intensity of the radiation field due to the increased active space the photon-emitted electron travels in.

In order to overcome these shortcomings (e.g., multiple matrix inversions) one may use some spectral method^22,23^, that on the other hand would imply additional convergence tests regarding the required number of basis functions for the correct dynamics; or, for instance, just a simple split-operator method, which however could not guarantee the stability during the temporal propagation. Hence, in order to avoid additional convergence tests, or multiple matrix inversions, and to ensure stability in the laser-induced dynamics, we considered the combination of the two methods: (i) the CN approach^24^ and (ii) the split-operator^25^ technique. According to this mixed approach (SOCN)^21^ the WF is divided into a $\Psi _0(z;t)$ field-free and a ${\tilde{\Psi }}(z;t)$ field-perturbed part: $\Psi =\Psi _0 + {\tilde{\Psi }}$. The solution in time moment $t_i+\Delta t$ of the stationary case ($i \partial _t \Psi _0 ={\hat{H}}_0 \Psi _0 =[{\hat{T}}+{\hat{V}}(z)] \Psi _0$) within the CN approach read as:

4 $$\begin{aligned} \Psi _0(z;t_i+\Delta t)\simeq e^{-i\Delta t {\hat{H}}_0}\Psi _0(z;t_i)\simeq {\hat{U}}^{\mathrm {CN}}_0 \Psi _0(z;t_i). \end{aligned}$$

For the field-perturbed part the following first-oder non-homogeneous linear differential equation (DE) can be deduced

5 $$\begin{aligned} i\partial _t {\tilde{\Psi }} = [{\hat{H}}_0+{\hat{V}}_{\mathrm {le}}(t)] {\tilde{\Psi }} + {\hat{V}}_{\mathrm {le}}(t)\Psi _0, \end{aligned}$$

which is generally solved by summing up a particular solution (${\tilde{\Psi }}_p$) and the general solution of the corresponding homogeneous DE, i.e., ${\tilde{\Psi }}_h(t+\Delta t)=\exp \{-i\int _{t}^{t+\Delta t}[{\hat{H}}_0+{\hat{V}}_{\mathrm {le}}(t')]\mathrm {d}t'\}{\tilde{\Psi }}_h(t)$ $\simeq \exp \{-i\Delta t V_{\mathrm {le}}(t+\Delta t/2)\}{\hat{U}}_0^{\mathrm {CN}}{\tilde{\Psi }}(t)$, where it is assumed that during the short $\Delta t$ time interval $V_{\mathrm {le}}(t)\simeq V_{\mathrm {le}}(t+\Delta t) \approx V_{\mathrm {le}}(t+\Delta t/2)$. In our approach, that considers this assumption, we deduced the solution of Eq. (5), first, by applying the CN scheme of the full WF:

6 $$\begin{aligned}&\Psi (t_i+\Delta t/2)=e^{-i\frac{\Delta t}{2}{\hat{H}}(t)}\Psi (t_i), \end{aligned}$$

7 $$\begin{aligned}&\Psi (t_i+\Delta t/2)=e^{i\frac{\Delta t}{2}{\hat{H}}(t+\Delta t)}\Psi (t_i+\Delta t). \end{aligned}$$

Then, by equalling Eqs. (6) and (7), and using $\Delta t_2=\Delta t/2$ along with the split technique

8 $$\begin{aligned}&e^{i\Delta t_2 {\hat{H}}_0} e^{i\Delta t_2 V_{\mathrm {le}}(t_i+\Delta t)} [\Psi _0(t_i+\Delta t)+{\tilde{\Psi }}(t_i+\Delta t) \nonumber \\& \quad \simeq e^{-i\Delta t_2 {\hat{H}}_0} e^{-i\Delta t_2 V_{\mathrm {le}}(t_i)} [\Psi _0(t_i)+{\tilde{\Psi }}(t_i)], \end{aligned}$$

the field-perturbed part in time step $t_{i+1}=t_i+\Delta t$ can be given as

9 $$\begin{aligned} {\tilde{\Psi }}(t_i+\Delta t)&=({\hat{U}}_0^{\dagger })^{-1} e^{-i \Delta t_2 V_{\mathrm {le}}(t_i+\Delta t)} [e^{-i\Delta t_2 V_{\mathrm {le}}(t_i)} \nonumber \\&\quad \,-\ e^{i\Delta t_2 V_{\mathrm {le}}(t_i+\Delta t)}] {\hat{U}}_0 \Psi _0(t_i) + {\tilde{\Psi }}_h(t_i+\Delta t) . \end{aligned}$$

After employing the first-order Taylor expansions of the exponentials inside the square brackets, and of the $\exp \{i\Delta t_2{\hat{H}}_0\}$ term (in *lhs* of Eq. (8)) one finally obtains

10 $$\begin{aligned} {\tilde{\Psi }}(z;t_i+\Delta t)&=({\hat{U}}_0^{\dagger })^{-1} \exp \left[ -i \Delta t_2 V_{\mathrm {le}}\left( z;t_i + \Delta t_2\right) \right] \nonumber \\&\quad \times \left[ -2i \Delta t_2 V_{\mathrm {le}}(z;t_i+\Delta t_2) \right] {\hat{U}}_0 \Psi _0\left( z;t_i\right) \nonumber \\&\quad +\exp \left[ -i \Delta t V_{\mathrm {le}}(z;t_i+\Delta t_2) \right] {\hat{U}}^{\mathrm {CN}}_0 {\tilde{\Psi }}(z;t_i), \end{aligned}$$

where ${\hat{U}}_0=[ {\hat{I}}-i \Delta t_2 {\hat{H}}_0(z)]$, and $({\hat{U}}_0^{\dagger })^{-1}$ is calculated by evaluating the inverse of the $[ {\hat{I}}+i \Delta t_2 {\hat{H}}_0(z)]^{-1}$ FD-represented matrix. In accordance with the CN scheme the field-free evolution operator is obtained by evaluating the ${\hat{U}}_0^{\mathrm {CN}}=({\hat{U}}_0^{\dagger })^{-1} {\hat{U}}_0$ matrix–matrix multiplication, while by adopting the split technique the external time-dependent field ($V_{\mathrm {le}}$) is detached from the full ${\hat{U}}^{\mathrm {CN}}$ operator. As a consequence, the inverse calculation of the evolution operator in each integration step is avoided, saving this way an appreciable amount of computation time. Using SOCN the ${\hat{U}}_0^{\mathrm {CN}}(\Delta t_j)$ matrices should be calculated only once, and stored in the computer’s memory at the very beginning of the simulation. Here $\Delta t_j=\Delta t_{\mathrm {init}}/2^{j-1}$ ($j=1,2, \dots$) represents the used discrete time intervals in the adaptive temporal propagation scheme ($\Delta t_1=10^{-3}$ at.u.).

### The initial state’s wavefunction and the solution of the TDSE

The initial state WF [$\Psi _0=\Psi (z;t=0)$] was calculated by the direct diagonalization of the field-free ${\hat{H}}_0={\hat{T}}+{\hat{V}}(z)$ Hamiltonian (large and sparse matrix) with the use of the Scalable Library for Eigenvalue Problem Computations (SLEPc) package^27,28^. Before initiating the TDSE runs (solving Eqs. (4), (10)), we determined the optimum value for the numerical discretization parameters: $\Delta z$ (gridpoints separation) and $\pm\, z_{\mathrm {max}}$ (grid-size). This was achieved using the SLEPc, and the value of the two discretization parameters was considered optimum when the calculated initial state WF converged. The SLEPc package can deliver that WF whose eigenenergy is the nearest to a user-given target eigenenergy. Since we considered for the active electron a surface electron, for the target eigenvalue the energy of $E_F+W$ was used. Once $\Psi _0(z)$ corresponding to the input energy was obtained, its accuracy was tested by defining the convergence parameter

11 $$\begin{aligned} d_{\mathrm {conv}}^{\Psi _0}(\Delta z_i)&= \Vert \Psi _0(\Delta z_{i}) - \Psi _0(\Delta z_{i-1}) \Vert \nonumber \\&\quad \times \Vert \Psi _0(\Delta z_{i-1}) \Vert ^{-1}, \end{aligned}$$

where $\ i\ge 1$, and for the initial value of $\Delta z_0$ we arbitrarily chose 0.8 at.u., while for $i>0$ values from $\Delta z_i \in \{0.7,0.6,0.5, \dots \}$. For the optimum gridpoint separation distance we fixed the value of $\Delta z=0.5$ at.u. when the $d_{\mathrm {conv}}^{\Psi _0}(\Delta z)<10^{-8}$ condition was fulfilled.

In order to diminish the undesired spurious reflections from the ’walls’ of the simulation box during the temporal propagation, and to monitor the extent part of the WF, we employed at edges of the simulation grid complex absorbing potentials (CAP)of the form^29^:

12 $$\begin{aligned} V_{\mathrm {CAP}}(z)= i \alpha _{\mathrm {absorb}}^2 \log \left[ \cos {\frac{z-z_{\mathrm {cut}}}{z_{\mathrm {max}}-z_{\mathrm {cut}}}}\right] ,\ (\mathrm {for}\ z>0) \end{aligned}$$

where $z_{\mathrm {cut}}$ is the first point of the absorbing region. The size of this region was $z_{\mathrm {max}}-z_{\mathrm {cut}}=50$ at.u. A similar CAP was used in the $z<0$ direction as well, however the absorption/reflection here is much less probable since the accelerating effect of the laser field inside the metal is not considered. By performing an initial testing for the case of the highest field strength (with peak intensity $I_0=80$ TW/$\hbox {cm}^2$) and longest pulse (3 optical cycles), we calculated the amount of the absorbed WF at these far distances, and noticed that by increasing the simulation space to $z_{\mathrm {max}}=1500$ at.u. ($\sim 79.3$ nm) in both directions ($z<0$ and $z>0$) the sum of the two cumulative absorbed WF norms stayed below the value of $10^{-10}$. This is an upper limit for all the considered laser intensities and pulse lengths, thus the full information of the system was maintained inside the simulated area. In this way a sufficiently large simulation ’box’ of $z\in [-\,1500, 1500]$ at.u. was constructed ensuring that even for the longest and the highest intensity pulse the relevant part of the ionized WF did not reach the boundaries (at $z= \pm \, z_{\mathrm {max}}$). In this way nonphysical simulation events that could significantly alter the characteristics of the final WF was prevented. During the integration of the TDSE in each time step the value of $\Delta t$ was initially set to $10^{-3}$ and later on adaptively modified (decreased iteratively by half) as long as the temporal propagation error defined as

13 $$\begin{aligned} d_{\mathrm {conv}}^{\Delta t}(t_i)&= \Vert \Psi (t_{i-1}+\Delta t/2) - \Psi (t_{i-1}+\Delta t) \Vert \nonumber \\&\quad \times \Vert \Psi (t_{i-1}+\Delta t) \Vert ^{-1}, \end{aligned}$$

exceeded the value of $10^{-8}$. However, since the calculation of $({\hat{U}}_0^{\dagger })^{-1}$ is a strongly time-consuming part of the simulation, in order to save even more CPU time we tried to reduce the number of required numerical matrix inversions during the complete temporal propagation as much as possible. This was achieved by using for the time-stepping $\Delta t= \frac{1}{2} \times 10^{-3}$, a value for which the total number of required inversions was kept acceptably low even for the case of the highest peak intensity (i.e., when the induced electron dynamics was changing in the fastest way).

## Photoelectron spectra and wavefunctions

By applying the aforementioned considerations in the solution of the TDSE, using the optimum values for the discretization parameters we solved Eqs. (4)–(10) for different peak intensity and central wavelength pulses in the single and few-cycle regime. As a result, the final electronic WFs ${\tilde{\Psi }}(z;t=\tau )$ and their associated photoelectron spectra (PhES) were calculated. The PhES were obtained by projecting the ionized (departed) part of the final wavefunction ${\tilde{\Psi }}^+(\tau )={\tilde{\Psi }}(z>0;\tau )$ onto the continuum states $\vert \psi _{\mathbf {k}}(\mathbf {z}) \rangle =(2\pi )^{-1/2}e^{ikz}$ (free plane waves) with kinetic momentum $\mathbf {k}$:

14 $$\begin{aligned} W_k(\tau )=\frac{\mathrm {d}P}{\mathrm {d}\mathbf {k}}=\left| \langle \psi _{\mathbf {k}}(\mathbf {z}) \right| {\tilde{\Psi }}^+\tau ) \rangle |^2, \end{aligned}$$

where $W_k(\tau )$ represents the ionization probability density at the end of the laser field.

**Figure 2:**
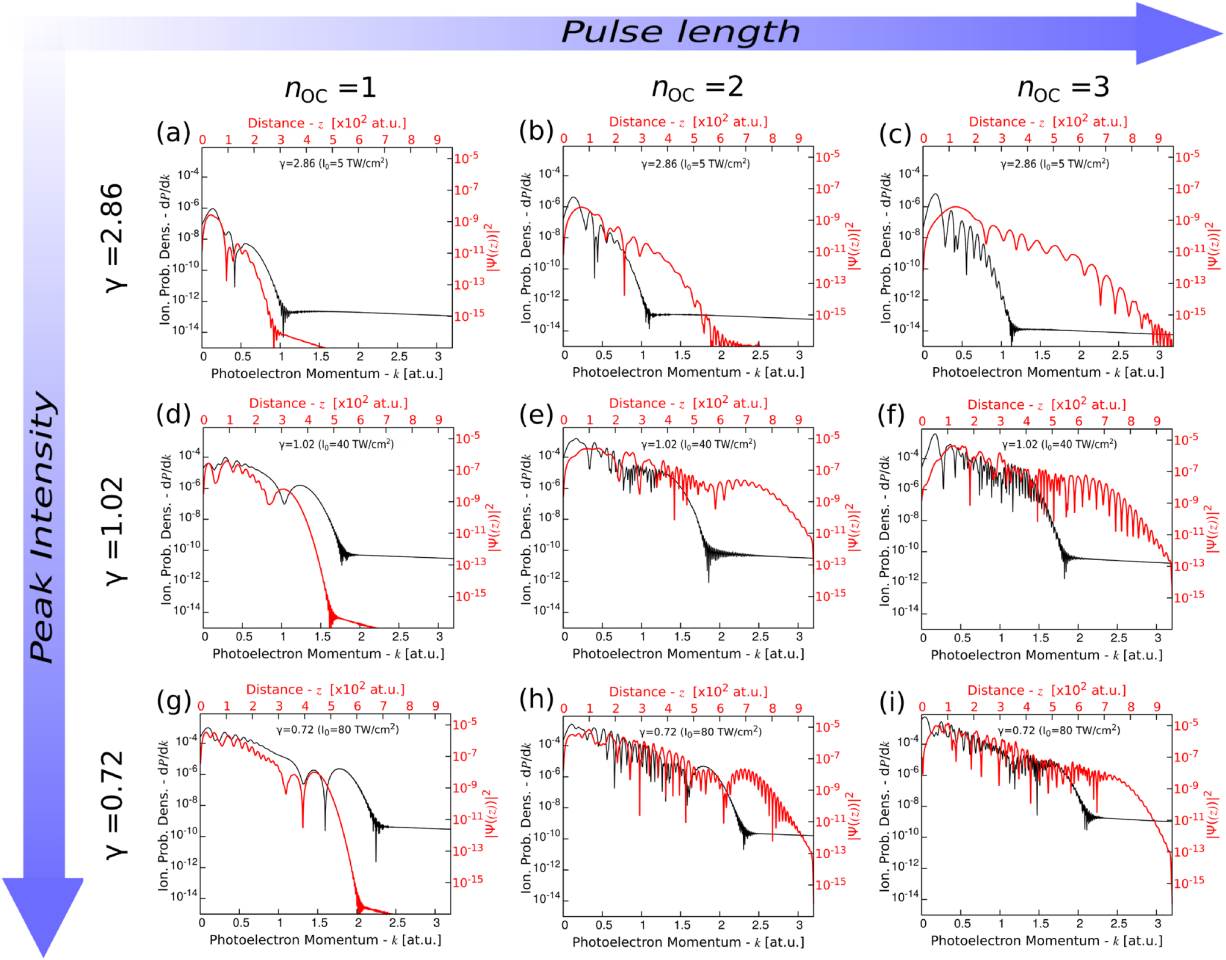
The final ($t=\tau$) electronic wavefunctions (red) and their corresponding photoelectron spectra (black) obtained in different photoemission regions: 1st row $\gamma _K = 2.8$ ($I_0 = 5\ \mathrm {TW/cm^2}$), 2nd row $\gamma _K = 1.02$ ($I_0 = 40\ \mathrm {TW/cm^2}$), 3rd row $\gamma _K = 0.7$ ($I_0 = 80\ \mathrm {TW/cm^2}$); and for different laser pulse lengths (number of optical cycles in the FWHM of $I_0$): 1st column $n_{\mathrm {oc}} = 1$, 2nd column $n_{\mathrm {oc}} = 2$, 3rd column $n_{\mathrm {oc}} = 3$ (one OC corresponds to $\tau = 2.66$ fs).

In the first step, we omitted the decaying amplitude of the plasmonic field enhancements and by fixing the field strengths through the whole coordinate space to $E(z;t)=E_0(t)$ we oriented our investigation onto the final dynamics obtained in the proximity of three different intensity regions: multiphoton $\gamma >1$ ($I_0 = 5$ TW/$\hbox {cm}^2$; $\gamma =2.8$), transition^30^ $\gamma \sim 1$ ($I_0 = 40$ TW/$\hbox {cm}^2$; $\gamma =1.02$), and strong-field $\gamma <1$ ($I_0 = 80$ TW/$\hbox {cm}^2$; $\gamma =0.72$) regime. We plotted in Fig. 2 the square of the final WF’s absolute value (thicker red curves) and the PhES (thinner black curves) calculated according to Eq. (14) in this three distinguished regimes for different pulse lengths: $n_{\mathrm {OC}}=1$, $n_{\mathrm {OC}}=2$, $n_{\mathrm {OC}}=3$ optical cycles at the FWHM of the intensity ($n_{\mathrm {OC}}=1$ corresponds to 2.66 fs).

As one may observe by increasing the incident field’s peak intensity, a plateau-like region of electrons start to build up in the PhES in the case of each pulse lengths. As long as for $\gamma =2.8$ the shape of the obtained spectra are more triangle-like, when the value of the Keldysh-parameter drops (intensity is increased) higher and higher energy (momenta) electrons start to appear with more or less the same probability in the middle region of the momentum maps: $1<k<1.5$ at.u. for $\gamma =1.02$ Fig. 2d–f, and $1.2<k<2$ at.u. for $\gamma =0.72$ Fig. 2g–i. The data show that these values do not depend on the pulse duration but exclusively on the strength of the driving field (observe the similar plateau for each intensity and different $n_{\mathrm {OC}}$). Moreover, as expected, the start and endpoint of this regions correspond to $2U_p$ and $10U_p$ energy values, respectively. $U_p=E_{\mathrm {loc},0}^2/4\omega _0^2$ is the ponderomotive (cycle-averaged quiver) energy of the photoelectron in the laser field, where $\omega _0=2\pi c/ \lambda$ represents the circular frequency and *c* is the speed of light. This means that upon $2U_p$ the directly ejected and accelerated electrons are present (predominantly for the case of $\gamma =2.8$), but by further increasing the pulse intensity higher energy electrons emerge in the PhES whose energy is greater than $2U_p$. This translates to the fact that a considerable portion of the ejected electrons are re-driven and accelerated back by the oscillating field onto the metal’s surface, where they collide with this by experiencing a field-modified potential. As the external field becomes reversed, the returning electron wave packet—provided that the potential-barrier’s distortion is strong enough—may experience a broaden and higher potential barrier (compared to the one when it was released in the continuum), and will undergo a forced and rapid deceleration phase. The newly emerged outward force due to the built-up barrier adds to the force exerted by the momentary field of the recently arrived optical half-cycle, and according to this the photoelectron will be accelerated even more in the outward direction due to the newly developed and increasing net force. In this way the electron may gain as much as $\sim 10U_p$ kinetic energy at the end of the optical half-cycle (and eventually at the end of the laser pulse itself). The presented data in Fig. 2 undoubtedly proves the presence of this secondary dynamics of electrons starting from $\gamma \approx 1$ and below 1. As seen, the increase of the pulse length does not have an important effect on this phenomenon, however since the more optical cycles are involved the more the electrons are ejected, and due to the increased number of electron wave packet trajectories interferences of these may recurrently occur bringing additional small wave-like features on top of the PhES.

While the cutoff energy is the same regardless of the pulse length (as already discussed previously), with the increase in the number of oscillating optical cycles the spectra show more and more sub-features, sub-components. These presumably correspond to above-threshold-photoemission (ATP) peaks, and one can see that for higher incident intensities, when the plateau starts to build-up in the PhES, this emerging plateau region is basically represented by a train of ATP peaks having about the same probability amplitude. The appearance of these wiggle-like features is even more straightforward by looking at the curves of the WFs: with the increase in the number of applied optical cycles the details are more obviously present. In addition to that, if one considers the shape of the WFs may note that for $n_{\mathrm {OC}}=1$ well distinguishable features/peaks can be identified, whereas by increasing $n_{\mathrm {OC}}$ next to the increased extent of the electron wave packets its higher complexity can also be observed.

Another observation is, that until a pulse is shorter and has a smaller number of ionizing optical cycles, the shape of the WF and PhES are more similar to each other, both having well distinguishable segments in their curves. On the other hand, the longer pulses will bring more discrepancies into this correspondence, mostly because the characteristics of the WF’s shape is slightly more affected by this than the shape of its momentum counterpart (i.e., the PhES), where by the integration over the space may smooth out some of the wiggle-like features present in the curves of the electrons wave packets. Nevertheless, since a fine correspondence between the shape of the ionized WF and PhES can be determined for ultrashort (single, few cycle) pulses, this could promise novel possibilities for evaluating and comparing experimentally acquired PhES by simply calculating and investigating the electron’s WF.

## Plasmonic effects and the cutoff law

In quite a many atomic physics paper a well known formula is used for defining the cutoff energy of the laser-ionized photoelectron’s, which reads as:

15 $$\begin{aligned} E_{\mathrm {cutoff}}=10U_p + 0.537W, \end{aligned}$$

where next to the $10U_p$ (with $U_p \sim I_0\lambda ^2$) a 0.537 W quantum correction term appears with *W* being the ionization energy of the atom. Contrary to photoemission from metals, this formula was deduced for atomic potential and external radiation field that was considered homogeneous throughout the space.

Certainly, in the present work the potential (Fig. 1a) is different from the atomic one and in the case when plasmonic effects in the vicinity of the target are also taken into account the radiation field loses its homogeneity as well. In order to check the applicability of the referenced formula (Eq. (15)) for the plasmonic metallic targets we performed several runs where the decaying profile of the plasmon-enhanced field was considered in the three ionization regimes (multiphoton, transition, and strong-field). Here we considered an exponential decay along the 0z direction of the local $E_{0,\mathrm {loc}}$ field by multiplying it with the $\exp \{-z/l_f\}$ factor, where $l_f$ represents the decay-length of the radiation field. The $\delta =l_f/l_q$ was introduced to quantitatively indicate the relation of the decay-length’s value to the amplitude of the quiver motion maintained by the laser field, $l_q=qE_0/m\omega ^2$.

**Figure 3:**
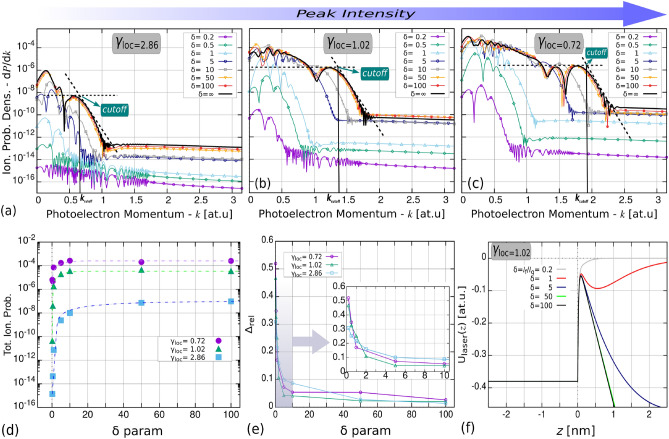
The final photoelectron spectra for different $\delta =l_f/l_q$ parameter and local Keldysh-parameter values (**a**) $\gamma _{\mathrm {loc}}=2.86$ [$I_{0,\mathrm {loc}} = 5\ \mathrm {TW/cm^2}]$, (**b**) $\gamma _{\mathrm {loc}} = 1.02$ [$I_{0,\mathrm {loc}}=40\ \mathrm {TW/cm^2}$], (**c**) $\gamma _{\mathrm {loc}}=0.71$ [$I_{0,\mathrm {loc}} = 80\ \mathrm {TW/cm^2}$]. The vertical solid lines indicate the positions of the cutoff momentum: (**a**) $k_{\mathrm {cutoff}}\simeq 0.6$, (**b**) $k_{\mathrm {cutoff}}\simeq 1.38$, (**c**) $k_{\mathrm {cutoff}}\simeq 1.91$, calculated from the $10U_p+0.537W$ formula^26^. In (**d**) the total ionization rates obtained for different Keldysh and delta paramater values. In (**e**) the relative differences between the spectra calculated for a certain $\delta <\infty$ to the PhES obtained when the field decay was absent ($\delta = \infty$) for the case of the three ionization regimes. Subfigure (**f**) illustrates the shape of the electron’s *V*(*z*) potential distorted by the maximum of the laser field for the cases of different $\delta$ decay-parameter values.

In Fig. 3a–c we plotted the final spectra obtained for a set of the field-decay parameter values: $\delta \in \{0.2, 0.5, 1, 5,$ $10, 50, 100 \}$; and also for the case when the laser-field of $n_{\mathrm {OC}}=1$ was considered homogeneous along the 0z direction at a constant value of $E(z;t)=E(z=0;t)=E(t)$, which corresponds to $\delta =\infty$. As one can see, irrespective of the local value of $\gamma$ in the PhES obtained for $\delta \le 1$ the ionization yields are low and can be characterized more with a triangle-like shape, i.e., given by the presence of the so-called *roll-off* electrons. Even in the case of the highest intensity where a large-scale plateau is already expected, only a short region of lower energy electrons are showing up with similar probability amplitudes. It is also obvious that as the value of $\delta$ becomes higher (the decay-length longer) the ionization probability density curves begin to rise until a final level is approached starting from $\delta \ge 5$. This observation can be noticed more clearly from Fig. 3d where the calculated ionization yields (the integral of d*P*/d*k* over *k* is performed) converge to the value obtained for $\delta =\infty$ homogeneous case. This convergence starts to take effect above $\delta =1$, and is slightly faster for smaller $\gamma$ values (higher intensities). By calculating the cutoff points given by Eq. (15) in each separate regime, we see that the expected cutoff momenta are attained only for $\delta > 10$, which proves that the cutoff formula derived for atomic targets and spatially homogeneous oscillating fields is valid only above $\delta \ge 10$, and can be safely used starting from $\delta \ge 50$ for 800 nm ultrashort IR pulses.

Since for the lower $\delta$ values the correct identification of the cutoff point is hard or even impossible to achieve, we compared the obtained spectra when $\delta \ne \infty$ to the case of the homogeneous field ($\delta =\infty$) by defining the following equation:

16 $$\begin{aligned} \Delta ^{(\delta )}_{\mathrm {rel}}=\frac{\int _0^{k_{\mathrm {max}}} |W_k^{(\delta )}(\tau ) - W_k^{(\delta =\infty )}(\tau )| \mathrm {d}k }{\int _0^{k_{\mathrm {max}}} W_k^{(\delta =\infty )(\tau )} \mathrm {d}k }, \end{aligned}$$

which quantity tends to 1 for $\delta \rightarrow 0$ and to 0 for $\delta \rightarrow \infty$. As one can see in Fig. 3e for $\delta \ge 5$ this quantity for each intensity cases is already below 0.1, however in subfigures (a–c) we can observe that the cutoff point is still relatively far from the $10U_p$ value. Only starting from $\delta \ge 50$ we see very similar final spectra and cutoff point for each regimes. Moreover, we can observe that for higher local field amplitudes ($\gamma _{\mathrm {loc}}=0.72$) the cutoff for $\delta =50$ is located further away from the $10U_p$ value than in the case of lower intensity ($\gamma _{\mathrm {loc}}=2.86$). This observation indicates that the decaying profile of the plasmon enhanced electric field has more impact on the final spectra when the incident field has higher amplitude.

The explanation of this finding is encoded in the relationship between the extent of the emitted electronic wave packet and the decay-length of the plasmonic field. For lower $\delta$ values the incident field’s effect gets suppressed for larger distances measured from the nanotarget surface, and the wave packets ionized in the first part of the pulses could reach far regions from where cannot be driven back by a fast decaying driving field, ruling out this way the recollision. Moreover, a more abruptly decaying field would transfer less energy for the motion of the electron, than a homogeneous constant field would do through the whole coordinate space. Another aspect constitutes in the lower yields obtained for the same local intensity but different decay-lengths. The reason why one gets smaller amount of ejected electrons after the completion of the laser pulse can be easily explained considering Fig. 3f, which shows the distortion suffered by the potential barrier at the top of the main/central optical half cycle. It is obvious that for lower $\delta$ parameter values the width of the field-distorted barrier is larger, hence the probability of ionizing an electron from the initial state drops. This translates to fewer electron-birth events in the continuum, decreasing in accordance with this the final ionization yields. However, in real-life scenarios it may happen that due to the field-disturbed surface even for the case of lower $\delta$ values the active electrons could reach levels of higher excited states before the ionization takes place. From this higher energy levels the probability amplitude of escaping a broader barrier is increased and the same the electron yields in the continuum. This may alter a bit the real convergence seen in Fig. 3d, however the position of the cutoff region wouldn’t be impacted much, since it is more affected by the driving field’s strength in the continuum, thus our statement regarding its dependence upon the value of $\delta$ can be considered to be still valid.

## Conclusions and outlook

In the first part of this work, we presented a computationally efficient method to study the laser-initiated dynamics from metallic nanotarget surfaces for a single active electron. The method was based on the direct solution of the one-dimensional Schrödinger equation represented on a finite-difference grid. We showed that by the diagonalization of the field-free Hamiltonian-matrix a set of (initial) eigenstates can be obtained at once at the start of our investigation. By considering the active electron initially on the Fermi-level we calculated the final wavefunction and ionization spectra resulting from the interaction with external (800 nm) laser fields of different durations and peak intensities, by covering the three intensity regimes: multiphoton, strong-field, and transition. It was shown that by increasing the incident field intensities and by entering more in the strong-field regime (with the Keldysh-parameter $\gamma \le 1$), a plateau of higher energy electrons started to appear in the calculated spectra (characterized by a traceable cutoff point, starting from which the highly energetic electrons start to disappear from the spectra). These are strong-field effects that results in a detectable amount of high energy electrons in the measurements that also encode information regarding the enhanced local fields in the close vicinity of plasmonic nanotargets. In the final part of the paper, we showed the applicability of a cutoff-law that was deduced for atomic potentials also for the case of photoemission taking place from plasmonic nanoobjects considering different field enhancement profiles (decay lengths). We showed that the referenced cutoff-law can be safely utilized for photoemission when the field-enhanced local fields have not so abrupt decrease ($\delta \ge 50$) in the near vicinity of the surface.
